# β cells keep bad epigenetic memories of palmitate

**DOI:** 10.1186/1741-7015-12-104

**Published:** 2014-06-23

**Authors:** Delphine Fradin, Pierre Bougnères

**Affiliations:** 1 Inserm U986, Pincus Building, Bicêtre Hospital, Paris Sud University, 94275 Le Kremlin Bicêtre, France; 2 AP-HP, Department of Pediatric Endocrinology, Bicêtre Hospital, Pôle I3E, Paris Sud University, rue du Général Leclerc, 94275 Le Kremlin Bicêtre, France

**Keywords:** pancreatic cells, fatty acids, palmitate, epigenetics, gene expression, type 2 diabetes, islets of Langerhans, obesity, DNA methylation

## Abstract

Palmitic acid, or hexadecanoic acid, a 16-carbon saturated fatty acid (FA), accounts for approximately 38% of the total circulating FA in lean or obese humans. In an article published in *BMC Medicine*, Hall *et al*. report that cultured islets from healthy donors, when exposed to palmitate, undergo changes in CpG methylation that are associated with modifications of expression in 290 genes. Their results provide a first look at the mechanisms used by the endocrine pancreas of humans to keep a durable genomic imprint from their exposure to FA that can influence gene expression and possibly cell phenotype in the long term. It is likely that such studies will help understand the epigenetic response of β cells to a disturbed metabolic environment, especially one created by obesity.

Please see related article: http://www.biomedcentral.com/1741-7015/12/103

## Background

In order to understand the findings of Hall *et al*. [1] one would have to ask why would β cells keep an epigenetic memory of prior exposure to fatty acids (FA)? The β cells continuously receive and integrate multiple neurosecretory, endocrine and metabolic signals originating mostly from the brain. Furthermore, they are also equipped to sense the circulating concentration of energy substrates such as glucose or FA produced by the liver and the adipose tissue, respectively. In contrast to glucose, plasma FA varies over a physiological ten-fold concentration range in response to the whole spectrum of ‘fight-or-flight; activities that were common in the early times of *Homo sapiens* during everyday life: strenuous or prolonged physical activity [2], protracted fasting, fear, psychological stress [3], cold exposure [4] and exercise in cold temperatures [5]. In each of these situations, an almost immediate increase in circulating FA up to approximately 2 mmol/L is caused by β-adrenergic stimulation of adipose tissue lipolysis. Lasting more than several hours, the increase in FA is intended to provide readily oxidizable fuels to skeletal muscle and heart and spare glucose for concomitant brain activity. Insulin secretion, a major player in this metabolic response, decreases in response to the secreted catecholamines. This decrease contrasts with the increased insulin secretion elicited by FA when they are infused alone, a situation where β cell activity is not shut down by catecholamines [6]. Palmitate is also increased in other circumstances of normal human physiology. The palmitate level increases during pregnancy [7] (but does not flow from mother to fetus) and during lactation. Birth is associated with massive palmitate release [8]. The human β cells are thus accustomed to facing transient increases in palmitate concentrations at times as part of normal life. It would be tempting to speculate that since these events are more than common, β cells would find little interest in keeping memories of such short-lived elevations of FA.

## Fatty acids from obesity to type 2 diabetes

What early human β cells are not used to facing is a protracted elevation of plasma FA that lasts for years and is not associated with catecholamine secretion. This is precisely the abnormal situation seen today in millions of humans accumulating fat and becoming obese, in whom mounting insulin resistance of the adipose tissue begets FA release from fat stores. While this is occurring, β cells synthesize and secrete abundant insulin, proliferate and enlarge [9]. Many factors have potential roles in the regulation of obesity-driven β-cell proliferation, including nutrients, insulin, incretins, hepatocyte growth factor, and recently identified liver-derived secreted factors. Much is still unknown about the proliferative pathways involved and the regulation of β-cell replication. Nonetheless, as the duration and/or the severity of obesity persist, beta cell mass decays and fails to compensate for insulin resistance. This, together with β-cell dysfunction, leads to the onset of type 2 diabetes (T2D). It is becoming evident that elements in insulin signal-transduction pathways are key to regulating β cell growth. Increased β cell apoptosis is an important factor contributing to β cell loss and the onset of T2D [10].

What is the basis of β cell failure in T2D? FA have long been shown to play a deleterious role, not only in peripheral tissues [11], but also in the endocrine pancreas by affecting the tenuous balance between effective pancreatic β-cell mass (through FA-induced lipotoxicity) and insulin resistance (which increases circulating FA) [12,13]. In contrast with the transient FA elevations occurring in normal life, when β cells cease their secretory activity, the sustained FA elevations of obese patients [14] or of non-obese patients with T2D [15] become part of the abnormal metabolic environment of β cells that are chronically overstimulated by both insulin resistance and hyperglycemia. At the β cell level this exposure to high FA levels and high insulin secretory activity by the pancreas represents a mismatch versus the evolutionary ‘natural’ situation.

## The epigenetics of lipotoxicity to the endocrine pancreas

To unravel the epigenetic effects of FA on β cells, Hall *et al*. [1] studied changes in DNA methylation at CpG nucleotides induced by 1 mmol/L palmitate in cultured human pancreatic islets. They found that palmitate slightly but significantly increased the level of DNA methylation (44.9% *versus* 43.9%) in most regions of the genome, including regions involved in the regulation of gene expression, such as promoters and CpG island shores or shelves, not CpG islands themselves. To those who are not familiar with the expression of methylation as a percentage, the finding means that when exposed to palmitate, approximately 1% more of the cultured β cells became methylated at the said genomic regions. Even if statistically significant, this is nonetheless a minor change at the level of the whole β cell population. More importantly, exposure to palmitate changed the degree of DNA methylation of 46,977 sites at the level of *P* <0.05, but none reached the significant false discovery rate (FDR) q-value of 0.05. A total of 4,561 sites, including 2,753 genes, displayed increased DNA methylation due to palmitate treatment while only 129 sites (94 genes) showed decreased DNA methylation. In this case, the changes in the islet cell population appear more biologically important, since 46% to 84% of the cells seemed to change their methylation status at specific gene positions due to palmitate treatment. Palmitate treatment of the cultured islets also altered glucose-induced insulin secretion and modified the expression of 1,860 genes that were down- or up-regulated. By integrating DNA methylation and gene expression data, 290 genes were found to show concomitant changes both in expression and in CpG methylation, most showing a decreased expression and an increase of DNA methylation. In cultured islets from donors affected by T2D, Hall *et al*. [1] observed that 37 of the previously found 1,860 genes showed changes in expression *versus* non-diabetic donors. Previous studies have already examined the effect of palmitate on gene expression in human islets [16,17] and it will be interesting to compare the results of the three studies to determine how consistent they are.

## Conclusions

The experimental system used by Hall *et al*. [1] to study DNA methylation is not ideal. First, islets in culture were 80% pure and were not composed uniquely of only β cells. Thus, some of the observed DNA methylation changes might reflect those occurring in non-β cells. Secondly, these islets are isolated from their normal physiological neuro-hormonal and metabolic signals. This may explain why cultured islets secrete less insulin when exposed to FA, while *in vivo* acute exposure of islets to FA is associated with increased insulin secretion in healthy subjects [6]. Thirdly, challenges exist also for the study of islets from donors with T2D, that tend to be much more sensitive to the isolation procedures compared to islets cultured from non-diabetic controls [17]. In addition, islets from donors with T2D may include cells from the inflammasome, possibly associated with T2D [18]. Nevertheless, the observation of palmitate-induced changes in DNA methylation paves the way for further defining the metabolic memory of β cells at an epigenetic level. In this respect, it would have been interesting to know if Hall *et al*. [1] found differentially methylated regions in islets from T2D donors *versus* non-diabetic islets, or if they have compared their methylation data with those collected by Dayeh *et al*. [19] and Volkmar *et al*. [20] for a combined analysis, one that is made mandatory by the small number of islet donors that can be analyzed by any individual investigator. Indeed, then, it is premature to comment further on the islet-expressed genes whose methylation status is altered by palmitate.

Instead of highlighting imperfections of the cultured islet system or lamenting the limited statistical relevance of a small number of pancreatic samples from heterogeneous groups of patients and controls, one should enjoy the preliminary insights provided by the epigenetic analysis in the master organ that drives the pathophysiology of T2D and consider the limitations as opportunities for additional research to clarify further the full extent of the clinical relevance of the tantalizing data provided by Hall *et al*. [1]. Clearly, this must be done since millions of modern obese humans continuously expose their β cells to an abnormal FA environment.

## Abbreviations

FA: fatty acids; FDR: false discovery rate; T2D: type 2 diabetes.

## Competing interests

The authors declare they have no competing interests.

## Authors’ contributions

Both authors contributed to the conception of the article. PB drafted the article. Both authors were involved in editing and revision of the manuscript and both authors read and approved the final manuscript.
